# New Concepts in Phospholipase D Signaling in Inflammation and Cancer

**DOI:** 10.1100/tsw.2010.116

**Published:** 2010-07-07

**Authors:** Julian Gomez-Cambronero

**Affiliations:** Department of Biochemistry and Molecular Biology, Wright State University School Medicine, Dayton, OH, USA

**Keywords:** phospholipase D (PLD), tyrosine kinases, GTPases, Grb2, S6K, mTOR, cell signaling, metastasis, chemotaxis, cell migration, inflammation

## Abstract

Phospholipase D (PLD) catalyzes the hydrolysis of phosphatidylcholine to generate the lipid second messenger phosphatidic acid (PA) and choline. PLD regulation in cells falls into two major signaling categories. One is via growth factors/mitogens, such as EGF, PDGF, insulin, and serum, and implicates tyrosine kinases; the other is via the small GTPase proteins Arf and Rho. We summarize here our lab's and other groups' contributions to those pathways and introduce several novel concepts. For the mitogen-induced signaling, new data indicate that an increase in cell transformation in PLD2-overexpressing cells is due to an increase of *de novo* DNA synthesis induced by PLD2, with the specific tyrosine residues involved in those functions being Y and Y. Recent research has also implicated Grb2 in tyrosine phosphorylation of PLD2 that also involves Sos and the ERK pathway. The targets of phosphorylation within the PLD2 molecule that are key to its regulation have recently been precisely mapped. They are Y, Y, and Y and the responsible kinases are, respectively, EGFR, JAK3, and Src. Y is an inhibitory site and its phosphorylation explains the low PLD2 activity that exists in low-invasive MCF-7 breast cancer cells. Advances along the small GTPase front have implicated cell migration, as PLD1 and PLD2 cause an increase in chemotaxis of leukocytes and inflammation. PA is necessary for full chemotaxis. PA enriches the localization of the atypical guanine exchange factor (GEF), DOCK2, at the leading edge of polarized neutrophils. Further, extracellular PA serves as a neutrophil chemoattractant; PA enters the cell and activates the mTOR/S6K pathway (specifically, S6K). A clear connection between PLD with the mTOR/S6K pathway has been established, in that PA binds to mTOR and also binds to S6K independently of mTOR. Lastly, there is evidence in the upstream direction of cell signaling that mTOR and S6K keep PLD2 gene expression function down-regulated in basal conditions. In summary, the involvement of PLD2 in cell signaling continues to expand geometrically. It involves gene transcription, mitogenic and cell migration effects as seen in normal growth, tumor development, and inflammation.

## INTRODUCTION

The present paper is both an update with our lab's recent results integrated with those from other labs working on phospholipase D (PLD) (particularly the PLD2 isoform) and a way of introducing novel concepts drawing from a general understanding of the field that can be directly applicable to cancer and inflammation. These new concepts comprise (1) a new model highlighting the regulation of PLD2 by phosphorylation-dephosphorylation and its implication in low-activity cancer cells, (2) a proposed novel model for the action of phosphorylated PLD2 on chemotaxis involving Grb2 and WASP, and (3) the introduction of the concept of mTOR/S6K upstream signaling and involvement on PLD2 gene expression.

PLD catalyzes the hydrolysis of phosphatidylcholine to generate the lipid second messenger phosphatidic acid (PA) and choline (Fig. 1). There are two mammalian isoforms: PLD1[1] and PLD2[2,3]. They share a highly conserved "HKD" domain necessary for catalysis[4]. PLD has been implicated in a variety of physiological cellular functions, such as intracellular protein trafficking, cytoskeletal dynamics, membrane remodeling and cell proliferation in mammalian cells, and meiotic division and sporulation in yeast[5,6,7,8,9]. PLD regulation in cells falls into two major signaling categories. One is by growth factors/mitogens, such as EGF, PDGF, insulin, and serum that implicate tyrosine kinases[2,10,11,12,13]; the other is by the small GTPase proteins Arf and Rho[14,15,16,17]. We shall review each of these signaling pathways providing the new findings that pertain to PLD.

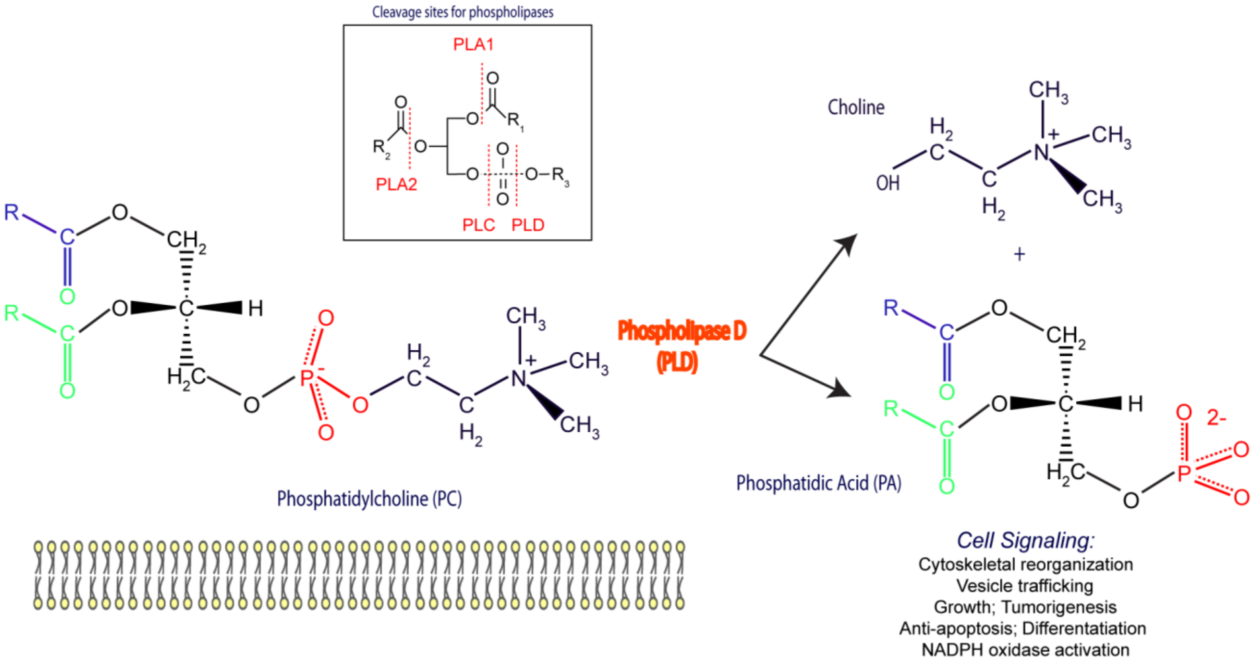

**FIGURE 1.** Substrate and products generated by the enzymatic action of PLD. The enzyme normally (albeit not exclusively) uses phosphatidylcholine that is hydrolyzed into choline and PA. The latter is a *bona fide* second messenger involved in many cellular functions (some included in the figure). The inset shows the sites of cleavage of PLD and related phospholipases in the glycerol backbone of the phospholipid substrate.

## PLD AND TYROSINE PHOSPHORYLATION AND DEPHOSPHORYLATION

PLD is activated in response to mitogenic signals, such as PDGF, EGF, FGF, insulin, IGF, VEGF, and serum[18,19,20,21,22,23,24,25]. The expression levels of the PLD gene are under developmental control and can also vary during the different stages of cell differentiation. Cell differentiation is accompanied by a very large increase in PLD2 expression. It has been demonstrated that PLD can also contribute to increased cell transformation[26]. Rat fibroblasts are transformed during elevated expression of Src or EGF-R and overexpression of PLD1 or PLD2[3,13,27]. Elevated PLD activity and tyrosyl-phosphorylated PLD2 have been reported in cells transformed by v-Src, v-Fps, v-Ras, and v-Raf[28,29,30,31,32]. The activation of this enzyme enhances cellular processes favorable for metastasis of EL4 lymphoma cells[33]. PLD also activates STAT3 by interacting with the thyroid oncogenic kinase RET/PTC[34]. PLD2 can form a complex with the EGF receptor[2] or with Pyk2 and Src kinases[35]. Hydrogen peroxide and EGF induce PLD phosphorylation and PKC-α activation[36], while activation of PLD by 8-Br-cAMP is accomplished through Src, Ras, and ERK[37].

Nevertheless, the signaling mechanisms that are in play for cell proliferation are still subject to current study. Our group has recently hypothesized that an increase in cell transformation in PLD2-overexpressing cells could be due to an increase of *de novo* DNA synthesis induced by PLD2. We uncovered an unexpected dual regulation by phosphorylation and dephosphorylation of lipase activity and proliferation-inducing capabilities of the human isoform PLD2, and have shown the specific tyrosine residues involved in those functions: $Y^{179}$ and $Y^{511}$[38].

## Gaps in Knowledge in Mitogen-Initiated PLD Signaling

Several gaps remain on this first “arm” of PLD regulation in spite of its importance in cell signaling. Although all studies agree with respect to tyrosine kinases in PLD signaling, different conclusions were drawn regarding lipase activity. For example, the isoform PLD1 can be phosphorylated on tyrosine, but this does not lead to changes in activity[39,40]. Also, whereas coexpression of the other isoform, PLD2, and Fyn or Fgr kinases leads to an enhancement of PLD activation and degranulation of mast cells[41], Ho et al.[11] have indicated that coexpression of Src and PLD1 results in an increased phosphorylation, but no increase in activity. Other authors have noted that the tyrosines $Y^{11}$, $Y^{14}$, $Y^{165}$, and $Y^{470}$ are important for PLD2 function[41], but, nevertheless, overexpression of the phosphorylation-deficient mutant, PLD2-Y11F, increases PLD2 activity in resting or EGF-activated cells[2].

Closely related to the enhancing effect of phosphorylation-deficient mutants, a new line of research has also implicated Grb2 in tyrosine phosphorylation of PLD2. In general, the phosphotyrosine motif *p-YxN* serves as a docking site for recruitment of SH2 domain–containing proteins, such as Grb2[11]. As indicated previously by our laboratory, PLD2 exists as a complex with Grb2 and PTP1B[42]. Also, Grb2 is found complexed with Sos *in vivo*[43,44,45], and PLD2 binds Grb2 and recruits Sos[46]. PLD-produced PA can bind directly to PLD[47] and it has been proposed that once activated, the cell could follow different paths alternatively during different stages of development: PLD2/Grb2/Sos and binding to Sos, either way resulting in the activation of Ras and the ERK pathway[48]. Tyrosine phosphatases also play a role in PLD regulation, as PTP1B coimmunoprecipitates with PLD2 and Grb2[49] through SH2 recognition sites on PLD2, which, in turn, bind to Sos[50,51]. To further connect PLD with the major pathways utilized by growth factors (Ras/MEK/ERK), Zhao et al.[48] demonstrated that PLD2-derived PA modulates Sos and Ras GTP/GDP exchange.

The target(s) of phosphorylation within the PLD2 molecule that are key to its regulation have not been precisely mapped. Our group set out to define the parameters of tyrosyl regulation of PLD2 by identifying the targeted amino acids that could explain a putative increase in enzymatic activity. We took advantage of the breast cancer cell line MCF-7, derived from a less-aggressive form of breast cancer that has relatively low levels of PLD activity. We identified three kinases capable of phosphorylating PLD2, EGFR, JAK3, and Src, and the phosphorylation sites as $Y^{296}$, $Y^{415}$, and $Y^{511}$, respectively. We provided evidence that explains PLD2 activity regulation by phosphorylation-dephosphorylation, and we further show that PLD2 activity is low in MCF-7 cells because it is kept down-regulated by tyrosyl phosphorylation on a specific “inhibitory” residue ($Y^{296}$) by a specific kinase, which we have identified as being EGFR (Fig. 2).

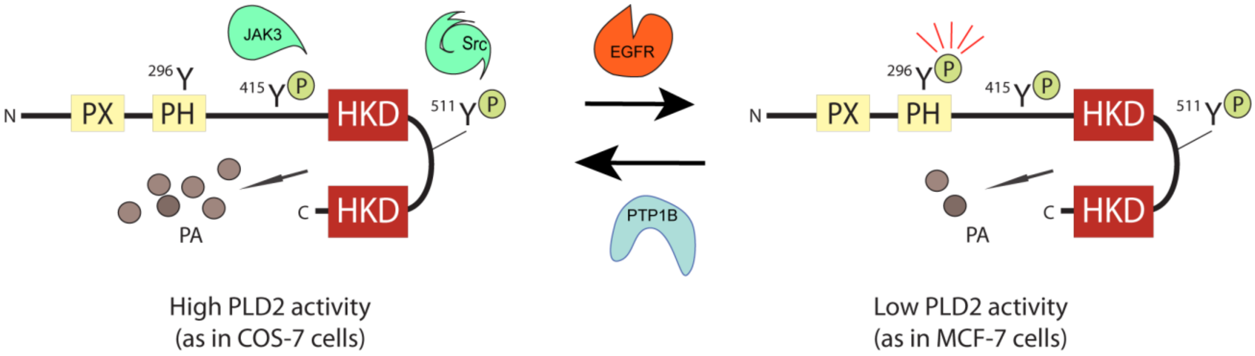

**FIGURE 2.** A new model highlighting the regulation of PLD2 by phosphorylation-dephosphorylation and its implication in low-activity cancer cells. Phosphorylation of $Y^{415}$ and $Y^{511}$ are essential for activity and are mediated by JAK3 and Src, respectively. Conversely, phosphorylation of residue $Y^{296}$ leads to a low-activity PLD2 (making it an "inhibitory" site), which is reversible by the action of PTP1B phosphatase. This has particular implications in cancer cells. PLD activity is frequently elevated in breast and other cancer cell lines, such as MDA-MB-231[118]. However, the MCF-7 cell line, derived from a less-aggressive form of breast cancer, has relatively low levels of PLD activity[119]. The Foster group has proposed that PLD induces the phenotype change from estrogen receptor positive (ER+) to estrogen receptor negative (ER–), which coincides with greater invasiveness of tumors, and that PLD is a signal for survival in serum-deprived cells[119,120,121]. In addition to this, we have proposed that a mechanism for a low-activity PLD2 to a high-activity PLD2 involves tyrosine phosphorylation of the inhibitory site $Y^{296}$ by EGFR kinase.

As indicated in the Introduction, two major signaling pathways are utilized by PLD. One is by tyrosine phosphorylation associated to its activation by mitogens and is the one just discussed. The second is by the small GTPase proteins Arf and Rho[14,15,16,17]. For this we have to talk about chemotaxis and inflammation.

## THE BIOLOGY OF CHEMOTAXIS AND ITS SIGNALING COMPONENTS

Chemotaxis is the complex physiological process comprising directional movement of cells towards a chemical stimulus. It has key biological implications that range from the simple migratory behavior of unicellular organisms towards nutrients to complex biochemical events, such as orchestration of components of an immune system to combat against infectious pathogens, tumor cells, sexual attraction of unicellular organisms[42], tissue organization, organogenesis, and homeostasis[52,53]. Lower-than-normal, excessive, or misdirected cell migratory behavior is considered to be the major pathological cause for several human diseases and disorders, such as asthma (eosinophils, mast cells), Crohn's disease (TH1 cells), multiple sclerosis (TH1 cells, monocytes), myocardial infarction (neutrophils), cardiac stroke (neutrophils), atherosclerosis (monocytes), rheumatoid arthritis (monocytes, TH1, neutrophils), type I diabetes (TH1, CD8), hepatitis (CD8), etc.[54]. A recent study from our lab indicated a clear involvement of either PLD1 or PLD2 in neutrophil chemotactic behavior[55].

Polymorphonuclear neutrophils (one subclass of leukocytes) participate in the body's first line of defense. Their directional migration from the blood capillary lumen to the site of chemical insult in peripheral tissues involves three primary steps: (1) rolling along the capillary wall endothelial cells, adhering to them through selectins, which are members of a family of cell adhesion molecules (CAM); (2) attachment to the endothelial cells through integrins, other members of the CAM family; and (3) diapedesis (i.e., transendothelial migration) from the capillary lumen to the site of injury. Although a wealth of research evidence suggests the role of numerous biochemical components, the exact mechanism of directional movement of neutrophils still remains a subject of intense study. Cell motility in leukocytes, such as neutrophils, involves the participation of small GTPases Rac and Cdc42, as well as ERK/ROCK and key proteins, such as WASP. As we shall discuss, chemotaxis also involves PLD.

## Activation of Rac

Receptor tyrosine kinase (RTK) activates PI3K which, in turn, activates Rac by complexing with endogenous Eps8, Abi1, and SOS-1, which act as Rac-specific GTP exchange factors (GEF)[56,57]. The recruitment of p85 to the trimeric Eps8-Abi1-SOS-1 complex with PI3K unmasks the autoinhibited Rac-GEF region of the complex. This GEF activity is further increased by the presence and recruitment of $PIP_3$, bolstering amounts of Rac-GTP to biologically significant levels. p85 and Abi1 colocalize to membrane ruffles on stimulation of RTKs and SH2 domain Ras activation. Rac activity is reliant on the presence of p85 and the activity of the GEF tricomplex[33].

## Cdc42 and Mena Complexes

Filopodia stimulation occurs as a result of activation of an IRSp53:Mena complex. IRSp53 contains a partial CRIB motif appropriate for Cdc42 binding. Upon binding, the Cdc42-GTP protein creates a conformational change in IRSp53 at the N-terminus, exposing the SH3 domain of IRSp53 to Mena for activation, initiating F-actin assembly in filopodia. Cell lines used to determine this experimentally include Swiss 3T3 for visualization of actin filaments and localized IRSp53:Mena complexes, and COS-7 for stable transfection of mutant Cdc42 and IRSp53 genes[57].

## WASp, N-WASP, and Associated Proteins

WASp and N-WASP are fully activated through Cdc42 and Rac, both in active GTP bound states. The WASp association with these two proteins along with the association with $PIP_2$ on the plasma membrane serves to release an autoinhibited N-terminal conformation of WASp and N-WASP, allowing association with Arp2/3 complex, leading to actin filamentation and filopodia formation[57]. N-WASP is also responsible for EGF endocytosis through clathrin-mediated pits, allowing the cell to internalize the EGF signals[58]. Actin accumulation is driven by the assembly of WASP family proteins and induces more efficient endocytosis of clathrin-coated pits, as actin fibers are nucleated in response to Rac-GTP.

## ERK-MAPK-ROCK and Motility Signals

Activated Rho compounds from PI3K pathways affect mDia and the ROCKI/II kinases initiated in focal adhesions. An example study shows that the ERK-MAPK pathway through Raf, a Ras effector, transcriptionally and post-transcriptionally controls cellular expression of Fra-1, a protein involved in regulating the function of RhoA, which subsequently influences the ROCKI/II and mDia proteins functional in cellular motility (actin filament capping and focal adhesion mechanisms). The Ras-Raf-Mek-Erk-AP-1 family cascade is responsible for this transcriptional regulation. Fra-1 has been cited to inhibit RhoA and therefore block both ROCKI/II kinases and capping protein mDia, leading to increased actin stress fibers and focal adhesion stability[59]. Another membrane-bound protein, uPAR, a urokinase-type plasminogen receptor, is dependent on the depression of RhoA by Fra-1 in order to ultimately activate Rac and drive polar lamellipodia formation[59]. As Fra-1 is highly expressed in colon tumorigenic cell lines, these Hct-116 and BE colon carcinoma cells are used for the determinations.

## PLD and Chemotaxis

Our laboratory and others have found a role for PLD in chemotaxis. We have proposed[33] the participation of PLD and Grb2 in cell chemotaxis involving three major pathways. First, the product of

PLD activity, PA, binds to target proteins mTOR, S6K, or Sos[48,60,61]. S6K then stimulates actin polymerization[62]. The residue $Y^{169}$ is involved in lipase activity (PA production), leading to chemotaxis. Second, PLD can bind to either Grb2 or Sos[43,63] with $Y^{179}$ involved in a PLD2-Grb2 protein-protein interaction that leads to a downstream activation of MAPK. MAPK can cross-talk to S6K and provide positive feedback[64] to enhance migration. In migration of LR5/RAW macrophages, S6K is mainly implicated through the residue $Y^{296}$. This residue is phosphorylated by EGFR kinase[65]. Third, PLD and PA can directly interact with actin[66,67] or, we hypothesize, through WASP (Fig. 3).

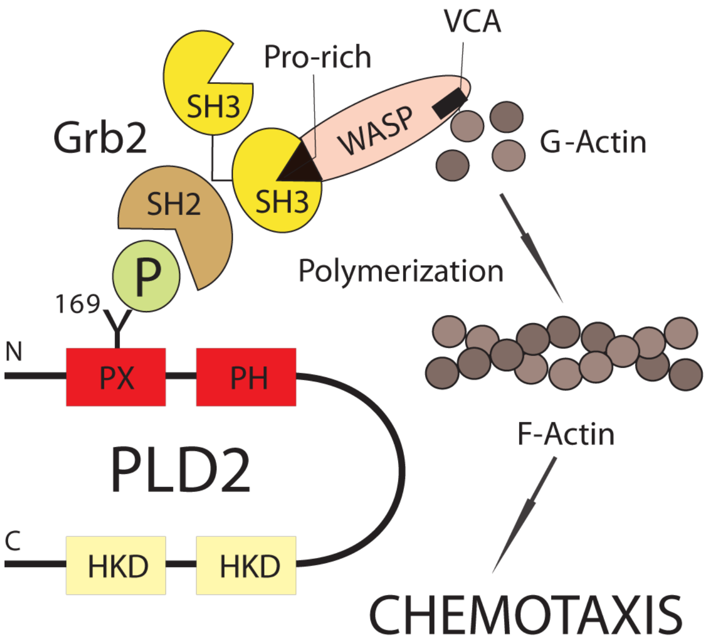

**FIGURE 3.** Proposed novel model for the action of phosphorylated PLD2 on chemotaxis involving Grb2 and WASP. One of the immediate actions of tyrosyl-phosphorylated, activated PLD2 is its association with Grb2, a docking protein that is known to serve as a nexus for other signaling proteins (Sos in the ERK pathway, etc.). Our laboratory demonstrated that Grb2 binds PLD2 at the $^{169}$**YLNR**$^{172}$ site through its SH2 domain. It is possible that Grb2 then becomes associated to the Wiskott-Aldrich syndrome protein (WASP) at its proline-rich region, through either of its two SH3 domains, forming a multimeric protein complex. WASP could lead to the potentiation of actin polymerization in FMLP- or IL-8–stimulated leukocytes.

## A Direct Role of PA on Cell Migration

In the process of chemotaxis, monomeric actin is polymerized to form F-actin (filamentous actin). The polymerization of actin filaments is tightly regulated through a variety of signaling pathways. One such pathway is the PI3K pathway. Phosphorylation of $PIP_2$ to $PIP_3$ by PI3K enables proteins with pleckstrin homology (PH) domains to associate to $PIP_3$ in the plasma membrane. One such PH-bearing protein is AKT, which, once recruited to the membrane, becomes phosphorylated by PDK1 and TorC2[68]. AKT is then able to associate with Rac and Cdc42, proteins involved in actin polymerization. The mTOR/S6K pathway is another PI3K effector, and mounting evidence indicates that it is involved in controlling motility in several cell types. As we shall discuss below, both molecules are known to bind to PA[69,70]. PA is a pleiotropic lipidic second messenger in yeast, plants, and mammalian cells. In the latter, it has been shown that vesicular trafficking and exocytosis are increased through promotion of membrane fusion by PA *in vivo*[71]. PA also causes the formation of transformed colonies in soft agar or tumors in xenografted nude mice of H-RasV12 oncogene-transfected fibroblasts[72]. PA induces calcium mobilization and chemotaxis through actin polymerization[73].

Proteins that are recruited or activated by PA have been identified in mammalian cells (reviewed in Jacinto and Hall[74]). Examples include Rac1, Fer kinase, the Ras exchange factor SOS, PI4P5Kinase, mTOR, PDK, S6K, Raf, Fgr, PKCζ, SHP, PP1, Arf kinesin, and the Phox-47 component of the NADPH oxidase of phagocytes. PA is generated in the cell by either the combined action of PLC and DGK[75], or directly by the action of PLD. PLD is a key signaling enzyme in stimulated platelets, neutrophils, mast cells, adipocytes, and certain cancer cells. In turn, PA can be metabolized to LPA and DAG, further enhancing the second-messenger prowess of PA in these mammalian cells[76].

Both PLD1 and PLD2 are involved in the process of leukocyte cell polarization[77], adhesion[78], and angiogenesis in zebrafish[79]. Recently, Nishikimi et al.[80] demonstrated that PA enriches the localization of the atypical GEF DOCK2 at the leading edge of chemoattractant-stimulated neutrophils as they begin to polarize and migrate. DOCK2-deficient neutrophils are unable to activate Rac2 in a polarized orientation and migrate in a directed manner. Thus, localized PA accumulation within the cell is crucial for the production of movement.

We have shown that PA is itself a chemoattractant that activates chemotaxis in a dose- and time-dependent manner[81]. We began that study by reasoning that the accumulation of PA in or near the inner leaflet of the cell membrane of budding lamellipodia could mimic a gradient of chemoattractant (or PA itself) outside the cell. We reported for the first time that extracellular PA serves as a neutrophil chemoattractant. As for the mechanism of how this would occur, we proposed and showed that PA enters the cell and activates the mTOR/S6K pathway (specifically, S6K), leading to cytoskeletal actin polymerization and chemotaxis. Similarly, we also demonstrated that cell-derived PA activates S6K and chemotaxis.

Let us review briefly the mTOR/S6K pathway to better understand the connection with PLD and its catalytic product, PA.

## THE mTOR/S6K SIGNALING PATHWAY

The “mammalian target of rapamycin” (mTOR) is also known in the literature as “FK506-binding protein” (FKBP12), “rapamycin-associated protein” (FRAP), “rapamycin and FKBP12 target” (RAFT1), “rapamycin target” (RAPT1), or “Sirolimus effector protein” (SEP). Every eukaryotic genome examined to date contains an mTOR gene[82]. The mTOR protein is highly conserved from yeast to man, sharing about a 95% homology at the amino acid level, indicating that it plays a vital role in cellular function[83]. Genetically, mTOR maps to the human chromosome 1p36.2[84].

### Cellular Importance of mTOR as it Relates to PLD

mTOR is known to sense mitogenic stimuli, extracellular nutrient levels[85], and ATP[86]. It also regulates cell functions, including actin reorganization of the cytoskeleton, cell growth and proliferation[87], cell survival[88], transcription, translation initiation[89], mRNA turnover, protein stability[90], and repression of autophagocytosis. Deregulation of the mTOR signaling pathway[91] is common in cancerous tumors, including lung, bladder, renal, ovary, breast, prostate, gastric, pancreatic, and head and neck carcinomas[92], and it has also been observed in lymphomas, melanoma, glioma, and several other brain diseases[93]. Unusually high mTORC1 activity is suspected as an underlying cause of these cancers[94].

Several labs have shown that PA production through PLD is an essential event for mitogenic activation of mTOR. Cellular concentrations of PA are normally less than 5% than that of phosphatidylcholine, but during mitogenic activation, the amount of PA within a cell increases, most likely due to PLD activation[95]. Using 0.3% 1-butanol, serum-stimulated PA production can be effectively stopped[96]. Using the same transphosphatidylation reaction, 1-butanol was able to block almost completely the serum-stimulated phosphorylation of S6K1 and 4E-BP1. The serum-stimulated activation of ERK1 and ERK2 was not affected by the presence of the 1-butanol. This experiment established the specificity of 1-butanol for PA production and implicates the involvement of PA in the phosphorylation of S6K and 4E-BP1[62]. From these findings, it can be asserted that PLD production of PA plays an essential role in the mTOR signaling pathway. The suppression of PA generated through PLD has been reported by a number of labs to inhibit the mTOR pathway in several cell phenotypes.

Chen's group has shown evidence that PA binds to mTOR at the FKBP12-rapamycin-binding (FRB) domain. Small unilamellar vesicles containing purified FRB fragment bound to PA in very low concentrations[95]. This binding was specific for PA, as other phospholipids were unable to bind the FRB with such specificity. The FRB region is where the rapamycin-FKBP12 molecule binds mTOR as well. It was hypothesized that the competition between the rapamycin-FKBP12 complex and PA for the FRB site may be one of the regulating factors in mTOR activation[62]. It was previously suggested that the rapamycin-FKBP12 molecule induces a conformational change in the structure of mTOR[96]. However, new data suggest that the effects of rapamycin are realized through the competitive interaction between mTOR's FRB and PLD-generated PA. Yet a third hypothesis suggests that the pH locally around mTOR is reduced by PA-generated PLD. mTOR is then prorogated in a way that promotes its kinase activity, or allows for interaction with yet unknown promoter substrates[97]. Recently, it has been demonstrated that PLD1 is an effector of the small GTPase Rheb within the mTOR signaling pathway[70,98].

Attempts to corroborate mTOR phosphorylation studies using exogenously provided PA have been inconclusive. Exogenously supplied PA was able to incorporate easily into cellular membranes and participate in cellular functions[98]; also, signaling was disrupted in siRNA PLD2 knockdown cells, even in the presence of exogenously provided PA[99]. Recently there have been some questions about the validity of studies involving exogenously provided PA. A few years ago, it was indicated that it is difficult for PA to cross cellular membranes and reach appropriate sites to engage mTOR, and exogenously provided PA may activate mTOR as a result of another, yet unknown pathway[83]. This pathway was recently discovered to be S6K[81].

## S6K and PLD

The best characterized function of mTOR is translation regulation[95]. The S6K isoform S6K1 is a major ribosomal protein in mammalian cells and is responsible for phosphorylating the S6 ribosomal protein[84]. The S6 subunit of the 40S ribosome selectively increases translation of some of the mRNA containing the 5'-terminal oligopyramidine tract (5'-TOP)[100]. mRNA with a 5'-TOP often serves to encode ribosomal proteins and other transcription regulation proteins, thereby up-regulating transcription[101]. S6K is independent of mTOR[102]; however, even low concentrations of rapamycin are thought to inhibit the phosphorylation activity of S6K[70]. While the actions of S6K are not well understood[103], S6K1 likely plays an important role in the regulation of cell cycle progression, cell growth, and cell proliferation[104].

The mTOR and PI3K pathways can phosphorylate at least eight sites on S6K. These phosphorylation events are known to require several mediator molecules, including PDK1, AKT, PKC, and the small G proteins Cdc42 and Rac1[104]. It is likely that there are other yet-unknown mediator molecules. Only the phosphorylation of T229 on S6K's activation loop of the kinase domain has been described[105]. The remaining phosphorylation sites, while identified, remain less well understood. Two phosphorylation sites, serine 371 and threonine 389, are known to be required for S6K activity[106]. Experiments show that mTOR can phosphorylate T389 directly *in vitro,* while the kinase responsible for S371 is not yet known[107]. However, incubation with rapamycin causes dephosphorylation of threonine 229 and serine 404, suggesting mTOR's involvement with those residues as well.

Additionally, evidence now shows that mTOR phosphorylation is mediated by p70 S6K[108]. This feedback mechanism phosphorylates SER-2448 on mTOR and does not appear to be controlled by the

AKT pathway. This may be a negative feedback mechanism[109]. Our laboratory has shown that PA binds to S6K independently of mTOR[61] (Fig. 4A). Further, extracellular PA or bacterial PLD-produced PA enter the cell and are also capable of activating S6K, leading to increased cell migration[81].

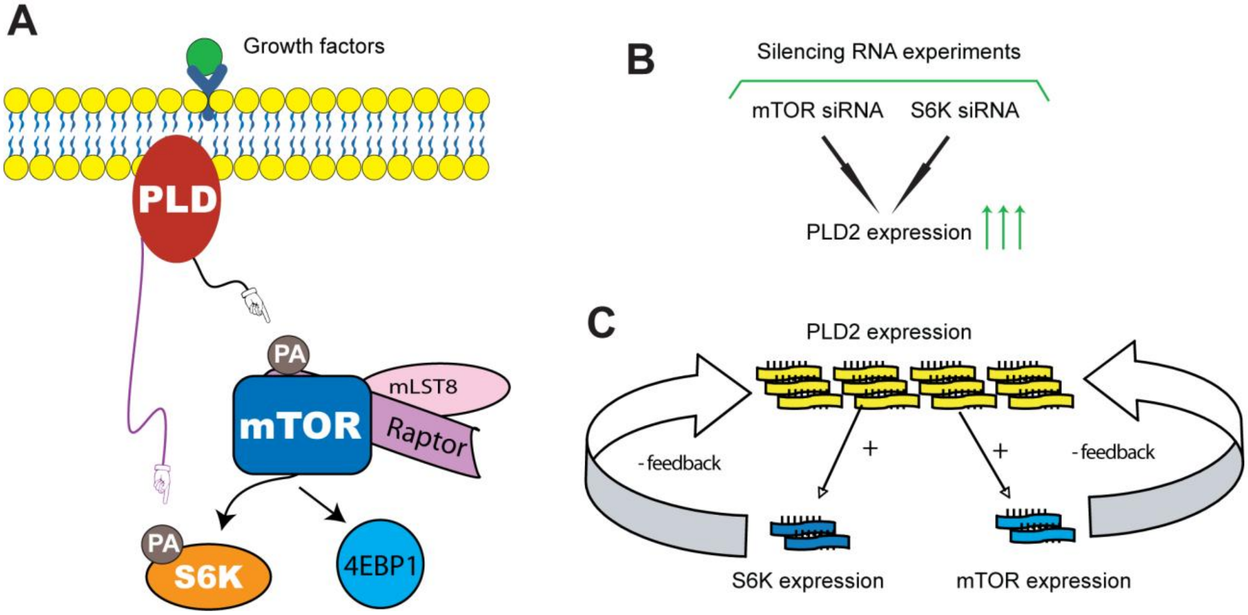

**FIGURE 4.** Introducing the concept of mTOR/S6K upstream signaling and involvement on PLD2 gene expression. (A) This panel shows that PLD produces PA, which directly binds to mTOR and also to S6K. mTOR (complex-1, associated to Raptor) activates S6K and that is the onset of protein translation in the cell and other functions. (B) Silencing experiments indicate that when S6K or mTOR are silenced, PLD2 gene expression is elevated. (C) Model showing that mTOR and S6K negatively regulate PLD2 expression. This can be rescued by cell stimulation with chemoattractants (such as IL-8 in leukocytes).

## CONNECTION OF PLD WITH KINASES AND GENE EXPRESSION

As just described, the impact of the mTOR/S6K signaling cascade is clearly important, especially considering that mTOR and/or S6K phosphorylate downstream targets resulting in the modulation of diverse cellular functions, such as survival, cell migration, growth, and proliferation[95,97,110,111,112]. Whether mTOR/S6K exerts an effect (positive or negative feedback) on other molecules, particularly PLD itself, has also been studied recently. While it is known that the product of PLD action, PA, activates the kinases mTOR and S6K, our laboratory has provided evidence for the first time in the upstream (or reverse) direction of cell signaling that mTOR and S6K keep PLD function down-regulated under basal conditions. These kinases do so by affecting the level of gene expression of PLD2 (Fig. 4B,C). Further, the cytokine IL-8 reverses this process and enables PLD to fully function as a facilitator for cell chemotaxis. This is important, as many tissues suffering from diverse pathological conditions are characterized by elevated IL-8.

The steady-state levels of any given mRNA is the result of both transcriptional and mRNA turnover (post-transcriptional) events. In leukocytes, mRNA turnover via regulation of mRNA stability accounts for almost half of all changes in mRNA levels[63]. Although the molecular aspects of PLD2's transcript up-regulation were not included in the aforementioned study, IL-8–mediated PLD2 up-regulation observed in HL-60 cells may be under post-transcriptional control. In line with this assumption is the fact that transcription of the PLD2 gene is a relatively slow process[113]. Further, IL-8 is known to induce genes involved in cell growth and migration via post-transcriptional mechanisms. Interestingly, mTOR

and S6K appear to be two of the many genes under post-transcriptional control in activated T lymphocytes[63].

Fast post-transcriptional changes of signaling protein concentrations (e.g., translation on demand) is a vital cellular process that ensures proper translation of stabilized transcripts at a given time[114]. Transient increases in PLD mRNA levels in response to the chemoattractant IL-8 may correlate with increased protein levels and/or PLD activity. It has been demonstrated that IL-8 increases migration in neutrophils and differentiated HL-60 cells in a PLD-dependent manner[115]. HL-60 cells were able to migrate towards a gradient of IL-8 following a similar kinetic pattern as the one for the PLD2/mTOR/S6K mRNA expression profiles. Moreover, IL-8 increases total PLD activity and this increase correlates with increased IL-8–mediated cell migration[116].

## CONCLUSIONS

PLD regulation in cells falls into two major signaling categories or "arms". One is via growth factors/mitogens, such as EGF, PDGF, insulin, and serum, and implicates tyrosine kinases; the other is via the small GTPase proteins Arf and Rho. We have summarized here our lab's and other groups' contributions to those pathways and introduce several novel concepts. The first "arm" involves Grb2, Sos, and the kinases EGFR, JAK3, and Src. The second "arm" involves GTPases and is directly related to chemotaxis, a process in which PLD plays a key role. While migration requires PLD2, mTOR, and S6K activities, mTOR/S6K functionality does participate in modulation of PLD2 function and expression. Further, the functional consequences of receptor activation are not limited to leukocyte locomotion, but also include degranulation, gene transcription, mitogenic and apoptotic effects, and are seen in angiogenesis, organogenesis, tumor development, growth, and metastasis[61,117]. The involvement of PLD2 in cell signaling continues to expand geometrically. It involves nearly all of the key cell biology functions: gene transcription, mitogenic and cell migration effects, as seen in normal growth, but also during tumor development and inflammation.

## ACKNOWLEDGMENTS

Dr. Cambronero's research is supported by grants HL056653 from the National Institutes of Health and the Ohio Board of Regents Research Challenge 667131. The author wishes to thank Stephen Short for his help with the references.

## References

1. Han, J.M., Kim, Y., Lee, J.S., Lee, C.S., Lee, B.D., Ohba, M., Kuroki, T., Suh, P.G., and Ryu, S.H. (2002) Localization of phospholipase D1 to caveolin-enriched membrane via palmitoylation: implications for epidermal growth factor signaling. *Mol. Biol. Cell* **13**, 3976.
2. Colley, W.C., Sung, T.C., Roll, R., Jenco, J., Hammond, S.M., Altshuller, Y., Bar-Sagi, D., Morris, A.J., and Frohman, M.A. (1997) Phospholipase D2, a distinct phospholipase D isoform with novel regulatory properties that provokes cytoskeletal reorganization. *Curr. Biol.* **7**, 191.
3. Lu, Z., Hornia, A., Joseph, T., Sukezane, T., Frankel, P., Zhong, M., Bychenok, S., Xu, L., Feig, L.A., and Foster, D.A. (2000) Phospholipase D and RalA cooperate with the epidermal growth factor receptor to transform 3Y1 rat fibroblasts. *Mol. Cell. Biol.* **20**, 462.
4. Pannequin, J., Delaunay, N., Darido, C., Maurice, T., Crespy, P., Frohman, M.A., Balda, M.S., Matter, K., Joubert, D., Bourgaux, J.F., Bali, J.P., and Hollande, F. (2007) Phosphatidylethanol accumulation promotes intestinal hyperplasia by inducing ZONAB-mediated cell density increase in response to chronic ethanol exposure. *Mol. Cancer Res.* **5**, 1147.
5. Exton, J.H. (2002) Regulation of phospholipase D. *FEBS Lett.* **531**, 58.
6. Lopez, I., Arnold, R.S., and Lambeth, J.D. (1998) Cloning and initial characterization of a human phospholipase D2 (hPLD2). ADP-ribosylation factor regulates hPLD2. *J. Biol. Chem.* **273**, 12846.

7. Gomez-Cambronero, J. (1995) Immunoprecipitation of a phospholipase D activity with antiphosphotyrosine antibodies. *J. Interferon Cytokine Res.* **15**, 877.
8. Brown, H.A., Henage, L.G., Preininger, A.M., Xiang, Y., and Exton, J.H. (2007) Biochemical analysis of phospholipase D. *Methods Enzymol.* **434**, 49.
9. Preininger, A.M., Henage, L.G., Oldham, W.M., Yoon, E.J., Hamm, H.E., and Brown, H.A. (2006) Direct modulation of phospholipase D activity by Gbetagamma. *Mol. Pharmacol.* **70**, 311.
10. Hess, J.A., Ross, A.H., Qiu, R.G., Symons, M., and Exton, J.H. (1997) Role of Rho family proteins in phospholipase D activation by growth factors. *J. Biol. Chem.* **272**, 1615.
11. Ho, W.T., Xie, Z., Zhao, Z.J., and Exton, J.H. (2005) Tyrosine phosphorylation of phospholipase D1 by v-Src does not per se result in activation. *Cell Signal.* **17**, 691.
12. Marcil, J., Harbour, D., Naccache, P.H., and Burgoin, S. (1997) Human phospholipase D1 can be tyrosinephosphorylated in HL-60 granulocytes. *J. Biol. Chem.* **272**, 20660.
13. Ahn, B.H., Kim, S.Y., Kim, E.H., Choi, K.S., Kwon, T.K., Lee, Y.H., Chang, J.S., Kim, M.S., Jo, Y.-H., and Min, D.S. (2003) Transmodulation between phospholipase D and c-Src enhances cell proliferation. *Mol. Cell. Biol.* **23**, 3103.
14. Brown, H.A., Gutowski, S., Moomaw, C.R., Slaughter, C., and Sternweis, P.C. (1993) ADP-ribosylation factor, a small GTP-dependent regulatory protein, stimulates phospholipase D activity. *Cell* **75**, 1137.
15. Cockcroft, S., Thomas, G.M., Fensome, A., Geny, B., Cunningham, E., Gout, I., Hiles, I., Totty, N.F., Truong, O., and Hsuan, J.J. (1994) Phospholipase D: a downstream effector of ARF in granulocytes. *Science* **263**, 523.
16. Bae, C.D., Min, D.S., Fleming, I.N., and Exton, J.H. (1998) Determination of interaction sites on the small G protein RhoA for phospholipase D. *J. Biol. Chem.* **273**, 11596.
17. Prelich, G. and Stillman, B. (1998) Coordinated leading and lagging strand synthesis during SV40 DNA replication in vitro requires PCNA. *Cell* **53**, 117.
18. Plevin, R., Cook, S.J., Palmer, S., and Wakelam, M.J. (1991) Multiple sources of sn-1,2-diacylglycerol in platelet-derived-growth-factor-stimulated Swiss 3T3 fibroblasts. Evidence for activation of phosphoinositidase C and phosphatidylcholine-specific phospholipase D. *Biochem. J.* **279,** 559.
19. Motoike, T., Bieger, S., Wiegandt, H., and Unsicker, K. (1993) Induction of phosphatidic acid by fibroblast growth factor in cultured baby hamster kidney fibroblasts. *FEBS Lett.* **332**, 164.
20. Song, J., Jiang, Y.W., and Foster, D.A. (1994) Epidermal growth factor induces the production of biologically distinguishable diglyceride species from phosphatidylinositol and phosphatidylcholine via the independent activation of type C and type D phospholipases. *Cell Growth Differ.* **5**, 79.
21. Seymour, L.W., Shoaibi, M.A., Martin, A., Ahmed, A., Elvin, P., Kerr, D.J., and Wakelam, M.J. (1996) Vascular endothelial growth factor stimulates protein kinase C-dependent phospholipase D activity in endothelial cells. *Lab. Invest.* **75**, 427.
22. Karnam, P., Standaert, M.L., Galloway, L., and Farese, R.V. (1997) Activation and translocation of Rho (and ADP ribosylation factor) by insulin in rat adipocytes. Apparent involvement of phosphatidylinositol 3-kinase. *J. Biol. Chem.* **272**, 6136.
23. Sa, G. and Das, T. (1999) Basic fibroblast growth factor stimulates cytosolic phospholipase A2, phospholipase C-g1 and phospholipase D through distinguishable signaling mechanisms. *Mol. Cell. Biochem*. **198**, 19.
24. Zhu, T., Ling, L., and Lobie, P.E. (2002) Identification of a JAK2-independent pathway regulating growth hormone (GH)-stimulated p44/42 mitogen-activated protein kinase activity. GH activation of Ral and phospholipase D is Src dependent. *J. Biol. Chem*. **277**, 45592.
25. Banno, Y., Takuwa, Y., Yamada, M., Takuwa, N., Ohguchi, K., Hara, A., and Nozawa, Y. (2003) Involvement of phospholipase D in insulin-like growth factor-I-induced activation of extracellular signal-regulated kinase, but not phosphatidylinositol 3-kinase or Akt, in Chinese hamster ovary cells. *Biochem. J.* **369**, 363.
26. Foster, D.A. and Xu, L. (2003) Phospholipase D in cell proliferation and cancer. *Mol. Cancer Res*. **1**, 789.
27. Joseph, T., Wooden, R., Bryant, A., Zhong, M., Lu, Z., and Foster, D.A. (2001) Transformation of cells overexpressing a tyrosine kinase by phospholipase D1 and D2. *Biochem. Biophys. Res. Comm.* **289**, 1019.
28. Song, J., Pfeffer, L.M., and Foster, D.A. (1991) v-Src increases diacylglycerol levels via a type D phospholipase-mediated hydrolysis of phosphatidylcholine. *Mol. Cell. Biol*. **11,** 4903.
29. Jiang, Y.W., Song, J., Zang, Q., and Foster, D.A. (1994) Phosphatidylcholine-specific phospholipase D activity is elevated in v-Fps-transformed cells. *Biochem. Biophys. Res. Commun*. **203,** 1195.
30. Carnero, A., Cuadrado, A., del Peso, L., and Lacal, J.C. (1994) Activation of type D phospholipase by serum stimulation and ras-induced transformation in NIH3T3 cells. *Oncogene* **9**, 1387.
31. Jiang, H., Lu, Z., Luo, J.Q., Wolfman, A., and Foster, D.A. (1995) Ras mediates the activation of phospholipase D by v-Src. *J. Biol. Chem*. **270,** 6006.
32. Frankel, P.A., Ramos, M., Flom, J., Bychenok, S., Joseph, T., Kerkhoff, E., Rapp, U.R., Feig, L.A., and Foster, D.A. (1999)Ral and Rho dependent activation of phospholipase D in v-Raf transformed cells. *Biochem. Biophys. Res. Commun.* **255**, 502.
33. Lehman, N., Di Fulvio, M., McCray, N., Campos, I., Tabatabaian, F., and Gomez-Cambronero, J. (2006) Phagocyte cell migration is mediated by phospholipases PLD1 and PLD2. *Blood* **108**, 3564.

34. Knoepp, S.M., Chahal, M.S., Xie, Y., Zhang, Z., Brauner, D.J., Hallman, M.A., Robinson, S.A., Han, S., Imai, M., Tomlinson, S., and Meier, K.E. (2008) Effects of active and inactive phospholipase D2 on signal transduction, adhesion, migration, invasion, and metastasis in EL4 lymphoma cells. *Mol. Pharmacol.* **74**, 574.
35. Banno, Y., Ohguchi, K., Matsumoto, N., Koda, M., Ueda, M., Hara, A., Dikic, I., and Nozawa, Y. (2005) Implication of phospholipase D2 in oxidant-induced phosphoinositide 3-kinase signaling via Pyk2 activation in PC12 cells. *J. Biol. Chem.* **280**, 16319.
36. Morris, A., Engebrecht, J., and Frohman, M.A. (1996) Structure and regulation of phospholipase D. *Trends Pharmacol. Sci.* **17**, 182.
37. Zhao, Y., Ehara, H., Akao, Y., Shamoto, M., Nakagawa, Y., Banno, Y., Deguchi, T., Ohishi, N., Yagi, K., and Nozawa, Y. (2000) Increased activity and intranuclear expression of phospholipase D2 in human renal cancer. *Biochem. Biophys. Res. Commun.* **278**, 140.
38. Henkels, K.M., Short, S., Peng, H.-J., DiFulvio, M., and Gomez-Cambronero, J. (2009) PLD2 has both enzymatic and cell proliferation-inducing capabilities, that are differentially regulated by phosphorylation and dephosphorylation. *Biochem. Biophys. Res. Commun.* **389**, 224.
39. Min, D.S., Kim, E.-G., and Exton, J.H. (1998) Involvement of tyrosine phosphorylation and protein kinase C in the activation of phospholipase D by H2O2 in Swiss 3T3 fibroblasts. *J. Biol. Chem.* **273**, 29986.
40. Min, D.S., Ahn, B.H., and Jo, Y.H. (2001) Differential tyrosine phosphorylation of phospholipase D isozymes by hydrogen peroxide and the epidermal growth factor in A431 epidermoid carcinoma cells. *Mol. Cells* **11**, 369.
41. Choi, W.S., Hiragun, T., Lee, J.H., Kim, Y.M., Kim, H.P., Chahdi, A., Her, E., Han, J.W., and Beaven, M.A. (2004) Activation of RBL-2H3 mast cells is dependent on tyrosine phosphorylation of phospholipase D2 by Fyn and Fgr. *Mol. Cell. Biol.* **24**, 6980.
42. Schlessinger, J. (1994) SH2/SH3 signaling proteins. *Curr. Opin. Genet. Dev*. **4**, 2530.
43. Horn, J., Lopez, I., Miller, M.W., and Gomez-Cambronero, J. (2005) The uncovering of a novel regulatory mechanism for PLD2: formation of a ternary complex with protein tyrosine phosphatase PTP1B and growth factor receptor-bound protein GRB2. *Biochem. Biophys. Res. Commun*. **332**, 58.
44. Chardin, P., Camonis, J.H., Gale, N.W., van Aelst, L., Schlessinger, J., Wigler, M.H., and Bar-Sagi, D. (1993) Human Sos1: a guanine nucleotide exchange factor for Ras that binds to GRB2. *Science* **260**, 1338.
45. Egan, S.E., Giddings, B.W., Brooks, M.W., Buday, L., Sizeland, A.M. and Weinberg, R.A. (1993) Association of Sos Ras exchange protein with Grb2 is implicated in tyrosine kinase signal transduction and transformation. *Nature* **363**, 45.
46. Li, N., Batzer, A., Daly, R., Yajnik, V., Skolnik, E., Chardin, P., Bar-Sagi, D., Margolis, B., and Schlessinger, J.(1993) Guanine-nucleotide-releasing factor hSosl binds to Grb2 and links receptortyrosine kinases to Ras signalling. *Nature* **363**, 85.
47. Di Fulvio, M., Lehman, N., Lin, X., Lopez, I., and Gomez-Cambronero, J. (2006)The elucidation of novel SH2 binding sites on PLD2. *Oncogene* **25**, 3032.
48. Zhao, C., Du, G., Skowronek, K., Frohman, M.A., and Bar-Sagi, D. (2007) Phospholipase D2-generated phosphatidic acid couples EGFR stimulation to Ras activation by Sos. *Nat. Cell Biol*. **9**, 706.
49. Hancock, J.F. (2007) PA promoted to manager. *Nat. Cell Biol*. **9**, 615.
50. Houle, M.G. and Bourgoin, S. (1999) Regulation of phospholipase D by phosphorylation-dependent mechanisms. *Biochim. Biophys. Acta* **1439**, 135.
51. Di Fulvio, M., Frondorf, K., Henkels, K.M., Lehman, N., and Gomez-Cambronero, J. (2007) The Grb2/PLD2 interaction is essential for lipase activity, intracellular localization and signaling in response to EGF. *J. Mol. Biol.* **367**, 814.
52. Koshland, D.E. (1982) Special topic: chemotaxis and motility. *Annu. Rev. Physiol*. **44**, 499.
53. Luster, A.D., Alon, R., and von Andrian, U.H. (2005) Immune cell migration in inflammation: present and future therapeutic targets. *Nat. Immunol*. **6,** 1182.
54. Ridley, A.J., Schwartz, M.A., Burridge, K., Firtel, R.A., Ginsberg, M.H., Borisy, G., Parsons, J.T., and Horwitz, A.R.(2003) Cell migration: integrating signals from front to back. *Science* **302,** 1704.
55. Vicente-Manzanares, M., Webb, D.J., and Horwitz, R.A. (2005) Cell migration at a glance. *J. Cell Sci*. **118**, 4917.
56. Innocenti, M., Frittoli, E., Ponzanelli, I., Falck, J.R., Brackmann, S.M., Di Fiore, P.P., and Scita, G. (2003) Phosphoinositide 3-kinase activates Rac by entering in a complex with Eps8, Abi1, and Sos-1. *J. Cell Biol*. **160**, 17.
57. Krugmann, S., Jordens, I., Gevaert, K., Driessens, M., Vondekerckhove, J., and Hall, A. (2001) Cdc42 induces filopodia by promoting the formation of an IRSp53:Mena complex. *Curr. Biol*. **11**, 1645.
58. Pollard, T.D. (2007) Regulation of actin filament assembly by Arp2/3 complex and formins. *Annu. Rev. Biophys. Biomol. Struct*. **36**, 451.
59. Benesch, S., Polo, S., Lai, F.P.L., Anderson, K.I., Stradal, T.E.B., Wehland, J., and Rottner, K. (2005) N-WASP deficiency impairs EGF internalization and actin assembly at clathrin-coated pits. *J. Cell Sci*. **118**, 3103.
60. Vail, E., Sahai, E., and Marshall, C.J. (2003) ERK-MAPK signaling coordinately regulates activity of Rac1 and RhoA for tumor cell motility. *Cancer Cell* **4**, 67.
61. Lehman, N., Ledford, B., Di Fulvio, M., Frondorf, K., McPhail, L.C., and Gomez-Cambronero, J. (2007) Phospholipase D2-derived phosphatidic acid binds to and activates ribosomal p70 S6 kinase independently of mTOR. *FASEB J*. **21**, 1075.
62. Fang, Y., Vilella-Bach, M., Bachmann, R., Flanigan, A., and Chen, J. (2001) Phosphatidic acid-mediated mitogenic activation of mTOR signaling. *Science* **294**, 1942.

63. Berven, L.A., Willard, F.S., and Crouch, M.F. (2004) Role of the p70(S6K) pathway in regulating the actin cytoskeleton and cell migration. *Exp. Cell Res*. **296**, 183.
64. Cox, D., Chang, P., Zhang, Q., Reddy, P.G., Bokoch, G.M., and Greenberg, S. (1997) Requirements for both Rac1 and Cdc42 in membrane ruffling and phagocytosis in leukocytes. *J. Exp. Med*. **186**, 1487.
65. Lehman, J.A. and Gomez-Cambronero, J. (2002) Molecular crosstalk between p70S6K and MAPK cell signaling pathways. *Biochem. Biophys. Res. Commun*. **293**, 463.
66. Henkels, K.M., Peng, H.-J., Frondorf, K., and Gomez-Cambronero, J. (2010) A comprehensive model that explains the regulation of phospholipase D2 (PLD2) activity by phosphorylation-dephosphorylation. *Mol. Cell. Biol.* **30,** 2251.
67. Kusner, D.J., Barton, J.A., Wen, K.K., Wang, X., Rubenstein, P.A., and Iyer, S.S. (2002) Regulation of phospholipase D activity by actin. Actin exerts bidirectional modulation of mammalian phospholipase D activity in a polymerization-dependent, isoform-specific manner. *J. Biol. Chem*. **277**, 50683.
68. Lee, S., Park, J.B., Kim, J.H., Kim, Y., Kim, J.H., Shin, K.J., Lee, J.S., Ha, S.H., Suh, P.G., and Ryu, S.H. (2001) Actin directly interacts with phospholipase d, inhibiting its activity. *J. Biol. Chem*. **276**, 28252.
69. Kamimura, Y., Xiong, Y., Iglesias, P.A., Hoeller, O., Bolourani, P., and Devreotes, P.N. (2008) PIP 3-independent activation of TorC2 and PKB at the cell's leading edge mediates chemotaxis. *Curr. Biol*. **18**, 1034.
70. Sun, Y., Fang, Y., Yoon, M.S., Zhang, C., Roccio, M., Zwartkruis, F.J., Armstrong, M., Brown, H.A., and Chen, J. (2008) Phospholipase D1 is an effector of Rheb in the mTOR pathway. *Proc. Natl. Acad. Sci. U. S. A*. **105**, 8286.
71. Corrotte, M., Chasserot-Golaz, S., Huang, P., Du, G., Ktistakis, N.T., Frohman, M.A., Vitale, N., Bader, M.F., and Grant, N.J. (2006) Dynamics and function of phospholipase D and phosphatidic acid during phagocytosis**.** *Traffic* **7**, 365.
72. Siddiqui, R.A. and English, D. (1997) Phosphatidic acid elicits calcium mobilization and actin polymerization through a tyrosine kinase-dependent process in human neutrophils: a mechanism for induction of chemotaxis. *Biochem. Biophys. Acta* **1349**, 81.
73. Buchanan, F.G., McReynolds, M., Couvillon, A., Kam, Y., Holla, V.R., Dubois, R.N., and Exton, J.H. (2005) Requirement of phospholipase D1 activity in H-RasV12-induced transformation. *Proc. Natl. Acad. Sci. U. S. A.* **102**, 1638.
74. Jacinto, E. and Hall, M.N. (2003) Tor signalling in bugs, brain and brawn. *Nat. Rev. Mol. Cell Biol.* **4**, 117.
75. Raghu, P., Manifava, M., Coadwell, J., and Ktistakis, N.T. (2009) Emerging findings from studies of phospholipase D in model organisms (and a short update on phosphatidic acid effectors). *Biochim. Biophys. Acta* **1791**, 889.
76. Topham, M.K. (2006) Signaling roles of diacylglycerol kinases. *J. Cell. Biochem.* **97**, 474.
77. Cummings, R., Parinandi, N., Wang, L., Usatyuk, P., and Natarajan, V. (2002) Phospholipase D/phosphatidic acid signal transduction: role and physiological significance in lung. *Mol. Cell. Biochem.* **234**, 99**.**
78. Powner, D.J., Payne, R.M., Pettitt, T.R., Giudici, M.L., Irvine, R.F., and Wakelam, M.J. (2005) Phospholipase D2 stimulates integrin-mediated adhesion via phosphatidylinositol 4-phosphate 5-kinase Igamma b. *J. Cell Sci.* **118**, 2975.
79. Zeng, X., Zheng, X., Xiang, Y., Plumley, H., Jessen, J.R., Zhong, T.P., Solnica-Krezel, L., and Brown, H.A. (2009) Phospholipase D1 is required for angiogenesis of intersegmental blood vessels in zebrafish. *Dev. Biol*. **328**, 363.
80. Nishikimi, A., Fukuhara, H., Su, W., Hongu, T., Takasuga, S., Mihara, H., Cao, Q., Sanematsu, F., Kanai, M., Hasegawa, H., Tanaka, Y., Shibasaki, M., Kanaho, Y., Sasaki, T., Frohman, M.A, and Fukui, Y. (2009) Sequential regulation of DOCK2 dynamics by two phospholipids during neutrophil chemotaxis. *Science* **324**, 384.
81. Frondorf, K, Henkels, KM, Frohman, MA, and Gomez-Cambronero, G. (2010) Phosphatidic acid is a leukocyte chemoattractant that acts through S6 kinase signaling. *J. Biol. Chem*. **285,** 15837.
82. Panwalkar, A., Verstovsek, S., and Giles, F.J. (2004) Mammalian target of rapamycin inhibition as therapy for hematologic malignancies. *Cancer* **100**, 657.
83. Crespo, J.L. and Hall, M.N. (2002) Elucidating TOR signaling and rapamycin action: lessons from Saccharomyces cerevisiae. *Microbiol. Mol. Biol. Rev*. **66**, 579.
84. Abraham, R.T. (2002) Identification of TOR signaling complexes: more TORC for the cell growth engine. *Cell* **111**, 9.
85. Kim, D., Sarbassov, D.D., Ali, S.M., et al. (2002) mTOR interacts with raptor to form a nutrient-sensitive complex that signals to the cell growth machinery. *Cell* **110**, 163.
86. Dennis, P.B., Jaeschke, A., Saitoh, M., Fowler, B., Kozma, S.C., and Thomas, G. (2001) Mammalian TOR: a homeostatic ATP sensor. *Science* **294**, 1102.
87. Martin, D.E. and Hall, M.N. (2005) The expanding TOR signaling network. *Curr. Opin. Cell Biol*. **17**, 158.
88. Hosoi, H., Dilling, M.B., Shikata, T., et al. (1999) Rapamycin causes poorly reversible inhibition of mTOR and induces p53-independent apoptosis in human rhabdomyosarcoma cells. *Cancer Res*. **59**, 886.
89. Gingras, A.C., Raught, B., and Sonenberg, N. (2001) Regulation of translation initiation by FRAP/mTOR. *Genes Dev*. **15**, 807.
90. Easton, J.B. and Houghton, P.J. (2006) mTOR and cancer therapy. *Oncogene* **25**, 6436.
91. Liu, L., Li, F., Cardelli, J.A., Martin, K.A., Blenis, J., and Huang, S. (2006) Rapamycin inhibits cell motility by suppression of mTOR-mediated S6K1 and 4E-BP1 pathways. *Oncogene* **25**, 7029.
92. Huang, S. and Houghton, P.J. (2003) Targeting mTOR signaling for cancer therapy. *Curr. Opin. Pharmacol*. **3**, 371.
93. Inoki, K. (2005) Dysregulation of the TSC-mTOR pathway in human disease. *Nat. Genet*. **37**, 19.
94. Buckland, A.G. and Wilton, D.C. (2000) Anionic phospholipids, interfacial binding and the regulation of cell functions. *Biochim. Biophys. Acta* **1483**, 199.
95. Fang, Y., Park, I.H., Wu, A.L., Du, G., Huang, P., Frohman, M.A., Walker, S.J., Brown, H.A., and Chen, J. (2003) PLD1 regulates mTOR signaling and mediates Cdc42 activation of S6K1. *Curr. Biol.* **13**, 2037.111.

96. Sun, Y. and Chen, J. (2008) mTOR signaling: PLD takes center stage. *Cell Cycle* **20**, 3118.
97. Foster, D.A. (2007) Regulation of mTOR by phosphatidic acid? *Cancer Res*. **67**, 1.
98. Fukami, K. and Takenawa, T. (1992) Phosphatidic acid that accumulates in platelet-derived growth factor-stimulated Balb/c 3T3 cells is a potential mitogenic signal. *J. Biol. Chem*. **267**, 10988.
99. Ha, S.H., Kim, D., Kim, I., et al. (2006) PLD2 forms a functional complex with mTOR/raptor to transduce mitogenic signals. *Cell Signal*. **18**, 2283.
100. Avruch, J., Belham, C., Weng, Q., Hara, K., and Yonezawa, K. (2001) The p70 S6 kinase integrates nutrient and growth signals to control translational capacity. *Prog. Mol. Subcell. Biol*. **26**, 115.
101. Kim, J.E. and Chen, J. (2000) Cytoplasmic-nuclear shuttling of FKBP12-rapamycin-associated protein is involved in rapamycin-sensitive signaling and translation initiation. *Proc. Natl. Acad. Sci. U. S. A*. **97**, 14340.
102. Meyuhas, O. (2000) Synthesis of the translational apparatus is regulated at the translational level. *Eur. J. Biochem*. **267**, 6321.
103. Hara, K., Yonezawa, K., Kozlowski, M.T., et al. (1997) Regulation of eIF-4E BP1 phosphorylation by mTOR. *J. Biol. Chem*. **272**, 26457.
104. Guertin, D.A. and Sabatini, D.M. (2007) Defining the role of mTOR in cancer. *Cancer Cell* **12**, 9.
105. Martin, K.A. and Blenis, J. (2002) Coordinate regulation of translation by the PI 3-kinase and mTOR pathways. *Adv. Cancer Res*. **86**, 1.
106. Alessi, D.R., Kozlowski, M.T., Weng, Q.P., Morrice, N., and Avruch, J. (1998) 3-Phosphoinositide-dependent protein kinase 1 (PDK1) phosphorylates and activates the p70 S6 kinase in vivo and in vitro. *Curr. Biol*. **8**, 69.
107. Pearson, R.B., Dennis, P.B., Han, J.W., et al. (1995) The principal target of rapamycin-induced p70s6k inactivation is a novel phosphorylation site within a conserved hydrophobic domain. *EMBO J*. **14**, 5279.
108. Burnett, P.E., Barrow, R.K., Cohen, N.A., Snyder, S.H., and Sabatini, D.M. (1998) RAFT1 phosphorylation of the translational regulators p70 S6 kinase and 4E-BP1. *Proc. Natl. Acad. Sci. U. S. A*. **95**, 1432.
109. Chiang, G.G. and Abraham, R.T. (2005) Phosphorylation of mammalian target of rapamycin (mTOR) at ser-2448 is mediated by p70S6 kinase. *J. Biol. Chem*. **280**, 25485.
110. Isotani, S., Hara, K., Tokunaga, C., Inoue, H., Avruch, J., and Yonezawa, K. (1999) Immunopurified mammalian target of rapamycin phosphorylates and activates p70 S6 kinase alpha in vitro. *J. Biol. Chem*. **274**, 34493.
111. Foster, D.A. (2009) Phosphatidic acid signaling to mTOR: signals for the survival of human cancer cells. *Biochim. Biophys. Acta* **1791**, 949.
112. Cheadle, C., Fan, J., Cho-Chung, Y.S., Werner, T., Ray, J., Do, L., Gorospe, M., and Becker, K.G. (2005) Control of gene expression during T cell activation: alternate regulation of mRNA transcription and mRNA stability. *BMC Genomics* **6**, 75.
113. Kikuchi, R., Murakami, M., Sobue, S., Iwasaki, T., Hagiwara, K., Takagi, A., Kojima, T., Asano, H., Suzuki, M., Banno, Y., Nozawa, Y., and Murate, T. (2007) Ewing's sarcoma fusion protein, EWS/Fli-1 and Fli-1 protein induce PLD2 but not PLD1 gene expression by binding to an ETS domain of 5' promoter. *Oncogene* **26**, 1802.
114. Bautista, M.V., Chen, Y., Ivanova, V.S., Rahimi, M.K., Watson, A.M., and Rose, M.C. (2009) IL-8 regulates mucin gene expression at the posttranscriptional level in lung epithelial cells. *J. Immunol.* **183**, 2159.
115. Beyer, A., Hollunder, J., Nasheuer, H.P., and Wilhelm, T. (2004) Post-transcriptional expression regulation in the yeast *Saccharomyces cerevisiae* on a genomic scale. *Mol. Cell. Proteomics* **3**, 1083.
116. Thelen, M. (2001) Dancing to the tune of chemokines. *Nat. Immunol.* **2**, 129.
117. Zheng, Y., Rodrik, V., Toschi, A., Shi, M., Hui, L., Shen, Y., and Foster, D.A. (2006) Phospholipase D couples survival and migration signals in stress response of human cancer cells. *J. Biol. Chem*. **281**, 15862.
